# Novel Approaches for Targeting Metalloproteinases

**DOI:** 10.3390/ph16121637

**Published:** 2023-11-22

**Authors:** Salvatore Santamaria

**Affiliations:** Department of Biochemical Sciences, School of Biosciences, Faculty of Health and Medical Sciences, Edward Jenner Building, University of Surrey, Guildford GU2 7XH, UK; s.santamaria@surrey.ac.uk

With 187 genes, metalloproteinases represent the most abundant protease family in the human proteome [1]. These proteases are involved in a variety of biological processes such as embryonic development, tissue resorption and repair, cell differentiation, migration, and apoptosis. As a result of this broad range of activities, dysregulated metalloproteinase activity is one of the drivers and hallmarks of diseases such as cancer, cardiac failure, atherosclerosis, and arthritis. The geometry of the active site around the catalytic ion, which is generally zinc, is highly conserved within each metalloproteinase superfamily, thus making it extremely challenging to achieve selective modulation of metalloproteinase activity.

This Special Issue aims to provide a state-of-the-art perspective on in vitro, preclinical and clinical approaches to modulate metalloproteinase activity for therapeutic purposes. Its 11 articles (5 reviews and 6 studies) cover a wide range of topics, from the screening of small molecules to tissue-specific drug delivery.

Hou et al. (contribution 1) performed ultrahigh-throughput activity assays (>650,000 molecules) to identify inhibitors of meprin α and β, two zinc metalloproteinases involved in several diseases such as cancer, fibrosis, and Alzheimer’s. The assay was based on the cleavage of a fluorescent peptide and optimized for a 1536-well plate format. Three different scaffolds (triazole-hydroxyacetamides, sulfonamide-hydroxypropanamides, and phenoxy-hydroxyacetamides) provided robust inhibition of meprin α, with good selectivity (>30-fold) over meprin β and other metalloproteinases. The most selective meprin α inhibitors contained hydroxamate as a zinc-binding group and therefore likely achieved their selectivity through optimized interactions with the protease subsites. Representative compounds were tested for their ability to affect the viability of skin fibroblasts and melanocytes. Little or no effect on cell viability was observed, suggesting a lack of cytotoxicity. Screening differently biased libraries or finely tuning assay conditions would aid the identification of non-chelating inhibitors. The lead compounds identified in this study were further optimized by Wang et al. using structure–activity relationship studies (contribution 2). The aryl triazole SR19855 exhibited a 10-fold selectivity for meprin α over meprin β and activity in the low-micromolar range. SR19855 was docked into a homology model of meprin α and β’s active site, thus highlighting crucial interactions between the protease and inhibitor. This highlighted that both the phenyl and pyrimidine rings of SR19855 could not be removed without a significant decrease in meprin inhibitory activity. The best compound, SR24717, exhibited sub-micromolar inhibitory activity against meprin α, with a 100-fold selectivity over meprin β, thus outperforming most of the previously reported meprin inhibitors and confirming the success of this approach. It will be interesting to probe the biological role of meprins by testing the effect of compounds like SR24717 in multiple cell-based assays.

High conservation of the active site across members of the metzincin superfamily has so far hampered the development of selective matrix metalloproteinase (MMP) inhibitors. The only MMP currently approved for clinical use is Periostat^®^ (doxycycline) for periodontitis. While monoclonal antibodies have achieved impressive results in terms of potency and selectivity [2], synthetic MMP inhibitors have failed in clinical trials due to lack of selectivity and poor pharmacokinetics [3]. To improve selectivity, hydrophilicity, and bioavailability, small molecule inhibitors have been modified through conjugation with carbohydrate moieties. These derivatives and their inhibitory profiles are widely discussed in the review by Cuffaro et al. (contribution 3). Carbohydrate-based compounds are a growing area in the metalloproteinase field that will likely generate new therapeutic opportunities in the near future.

Das et al. (contribution 4) comprehensively review alternative mechanisms for inhibiting MMP activity. These include targeting distantly located, poorly conserved substrate-binding sites (exosites), homodimer formation (in the case of MMP9 and MMP14), and zymogen activation. Selective inhibition can be achieved using peptides, small molecules, or monoclonal antibodies. The authors highlight that the MMP substrate repertoire is not limited to extracellular matrix (ECM) substrates, including, for example, chemokines and cytokines. To uncover the metalloproteinase degradome, proteomics techniques such as terminal amine isotopic labeling of substrates (TAILS) have been developed.

Gonçalves et al. (contribution 5) explore current approaches to target MMP2 in heart failure (HF). MMP2 is involved in the degradation of the cardiac ECM and components of the contractile sarcomeric apparatus such as troponin I, titin, and myosin light chain. Plasma MMP2 is considered a biomarker of HF, and rodent models have shown a link between dysregulated MMP2 activity and cardiac dysfunction. Starting from non-selective, first generation, zinc-chelating hydroxamate MMP inhibitors, the authors move to describe non-hydroxamate inhibitors, antibiotics of the tetracycline class, siRNA, statins, and antihypertensive drugs, as well as their applications in preclinical and clinical models of HF.

Skrzypiec-Spring et al. (contribution 6) tested the effect of β-blockers carvedilol, nebivolol, and metoprolol in a rat model of ischemia–reperfusion (IR) injury. These molecules were previously reported to inhibit expression of MMP2 [4,5]. IR induced activation of MMP2, which was reversed specifically by carvedilol but not by the other β-blockers, suggesting that the cardioprotective activity of carvedilol is partly mediated by inhibition of MMP2 activation. Supporting this, carvedilol caused a 73% decrease in the cardiac levels of the sarcomeric protein troponin I at the end of perfusion, a proxy for troponin I degradation. The exact mechanism of carvedilol action still warrants further investigation, but based on the results from the authors, no direct effect on protein expression seemed to be involved.

Palladini et al. (contribution 7) tested the effect of obeticholic acid (OCA) in a rat model of hepatic IR to assess modulation of MMP activity, since previous in vitro studies have shown that OCA can restore the balance between MMPs (in particular MMP2 and MMP9) and their endogenous inhibitors, the tissue inhibitors of metalloproteinases (TIMPs) and reversion-inducing cysteine-rich protein with kazal motifs (RECK) [6]. In the kidney cortex and medulla, OCA treatment resulted in reduced MMP9 dimer activity compared with vehicle-treated IR rats, but no significant changes in MMP2, TIMP1, TIMP2, or RECK were observed. Additionally, OCA reduced serum levels of creatinine, an index of renal function, and reduced levels of the proinflammatory cytokines tumor necrosis factor (TNF)-α and interleukin-6 (IL-6) in the kidney cortex. Overall, the results of this study provide evidence that OCA treatment may ameliorate hepatic renal syndrome by inhibiting fibrosis and restoring renal function.

Ciccone et al. (contribution 8) review the ability of natural compounds to modulate the activity of gelatinases MMP2 and MMP9 in the context of neurodegenerative and neuroinflammatory diseases, in particular Alzheimer’s disease. After summarizing the pathological and physiological role of MMP2 and MMP9 in the nervous system, the authors describe bioactive drugs derived from marine organisms and their application as gelatinase inhibitors. They then move on to discuss molecules derived from terrestrial sources, highlighting crucial differences in the mode of action, i.e., direct versus indirect (for example, transcriptional) inhibition.

Laghezza et al. (contribution 9) identified bisphosphonic acid derivatives as MMP13 inhibitors. These molecules can be tested for the treatment of bone metastasis due to the ability of the bisphosphonic acid group to specifically target the bone. The most potent compounds exhibited activity in the low/sub-micromolar range against MMP13 and good selectivity over MMP8 and MMP9. However, selectivity over MMP2 still needs to be improved.

Compared to MMPs, not much is known on the biological functions of dipeptidyl peptidase III (DPP III), a zinc-dependent exopeptidase. Agić et al. (contribution 10) used a combination of in vitro and in silico approaches to identify DPP III inhibitors. They reported coumarin-based compounds with inhibitory activity in the micromolar range that can be used to probe the patho-physiological role of DPP III. Quantitative structure activity relationship analysis identified crucial substituents necessary for inhibitory activity. The most active compound was docked in the active site of DPP III to model crucial interactions with the enzyme.

Metalloproteinases are also involved in osteoarthritis, the most common degenerative joint disease, due to their ability to degrade important ECM components such as collagens and proteoglycans. The lack of disease-modifying drugs prompted the development of innovative approaches to delay/arrest disease progression, as discussed in the review by McClurg et al. (contribution 11). Such strategies either aim to inhibit cartilage degradation (via their action on metalloproteinases such as A Disintegrin-like and metalloproteinase with thrombospondin motif 5 or MMP13) or promote cartilage anabolism (through administration of growth factors). The authors further described and critically discussed a number of methods to specifically target drug delivery to the cartilage, a poorly vascularized tissue, for example, through conjugation with peptides directed against chondrocytes or specific ECM components such as type II collagen or aggrecan.

The studies described in this Special Issue will undoubtedly stimulate further research in this area, thus increasing the druggability of metalloproteinases.
